# Effect of Fluid Media on Material Removal and Subsurface Defects Evolution of Monocrystal Copper in Nano-Cutting Process

**DOI:** 10.1186/s11671-019-3065-0

**Published:** 2019-07-17

**Authors:** Quanlong Wang, Chaofeng Zhang, Meiping Wu, Jiaxuan Chen

**Affiliations:** 1 School of Mechanical Engineering, Jiangnan University, Wuxi, 214122 People’s Republic of China; 2 Jiangsu Key Laboratory of Advanced Food Manufacturing Equipment &Technology, Wuxi, 214122 People’s Republic of China; 3 Center for Precision Engineering, Harbin Institute of Technology, Harbin, 15001 People’s Republic of China

**Keywords:** Nano-cutting, Fluid media, Materials removal, Subsurface defects evolution, Crystal structural transformation

## Abstract

The effect of fluid media on material removal and subsurface defects evolution in nano-cutting process of single-crystal copper is investigated by means of molecular dynamics simulation. In this paper, the removal mechanism of the chip and formation mechanism of machined surface are investigated by analyzing the atomic migration and dislocation evolution of workpiece during nano-cutting process with the use of aqueous media. The distribution of temperature and subsurface defect crystal structural transformation are investigated, which are analyzed by centro-symmetry parameter and common neighbor analysis methods. The results show that the workpiece material is removed by the extrusion shearing action of the cutting tool. The lubrication of the aqueous media can reduce the cutting force and lower the height of cutting chip. Particularly, the cooling action of the fluid media results in the formation of a typical defect “similar-to-grain boundary” in subsurface of the workpiece. And the temperature of workpiece has a distinct decrease during nano-cutting process with the use of fluid media.

## Background

Diamond cutting is widely considered to be an effective technique for the generation of nano-structured surfaces with submicron dimensional accuracy and nanometric surface finish [1]. The mechanism of material removal and formation of machined surface at the nanoscale is critical for the improvement of machining accuracy in nanometric cutting process. In early studies, the researchers focused on the deformation of the material [2–4], the surface defect evolution [5–7], the residual stress [8, 9], and the crystal phase transition [10, 11] during nano-cutting process in vacuum environment. However, in actual nanometric cutting of metal material, the role of cooling and lubricating fluid is very crucial for improving the performance of machining [12]. Therefore, it is necessary to study the influence of fluid media on the mechanism of material removal and subsurface defect evolution in nano-cutting process.

In recent years, the machining mechanism of nano-cutting process is widely investigated by molecular dynamics (MD) simulation which is proved to be an effective method to study nanometric cutting process. For instance, the effect of the recovery and side flow on the surface generation, and phase transformation in nano-cutting, is studied by Fang [13]. And it is found that the suppression of the side flow is an effective way to improve the generated surface roughness in nano-cutting. Urbassek [14] adopted MD simulation to study scratching of nanocrystalline metals and found that grain orientation generates a dominant effect on the pile-up shape and also influences the friction coefficient strongly. Sharma [15] investigated the effect of six different crystal orientations on material deformation mechanism, subsurface defects, cutting forces, specific cutting energy, ploughing effect, and surface roughness in nanoscale cutting. Luo [16] demonstrated the shape transferability by using nanoscale multi-tip diamond tools in the diamond turning for scale-up manufacturing of nanostructures.

The subsurface defect formation and the surface roughness of the nano-component can be restrained by reasonable selection of cutting parameters, such as cutting direction, cutting depth, cutting speed, and tool geometry. However, it cannot succeed in inhibiting the formation of surface defects and improving the surface quality from generation mechanism. Besides, these researches are focused on nano-cutting process in vacuum environment. Actually, in nanometer cutting, atmosphere and cooling liquid media existed between tool and workpiece, which can affect the nano-cutting mechanism and the surface quality of nanostructure.

Based on the above consideration, many scholars carried out the research on nano-machining process with the use of fluid media. For example, Mylvaganam [17] explored the effect of O_2_ on the nano-indentation of diamond cubic silicon using MD simulation and found that the O_2_ molecule dissociates into oxygen atoms and forms chemical bonds with silicon atoms. Rentsch [18] found that cutting fluid makes large effect on the distribution of stress and temperature and pointed out that the cutting fluid can reduce the tool wear. Liu [19] studied the influence of atmospheric molecules on surface quality and tool wearing in nano-cutting. The results indicated that the cutting force decrease and the wearing of tool is reduced on account of the lubrication of atmospheric molecules. Singh [20] researched the influence of nano-particle cutting fluid on metal removal process. Wang [21] discussed the effect of water molecules on tribological behavior and property measurements in nano-indentation processes and found that the participation of water molecules makes the initial indentation force increases and the biggest indentation force decrease. Chavoshi [22] studied high-temperature nanoscratching of single-crystal silicon under reduced oxygen condition, and no remnants of high-pressure silicon phases were observed in the simulation.

From the available literatures, previous researches about nano-cutting process using fluid media are based on the simulation models of few molecules or nano-particles, which is located in the area of tool-chip interface. However, no fluid media is added in other areas, and the lubrication of fluid media is analyzed restrictedly. Due to the insufficiency of fluid media in the models, the cooling action of the fluid media does not affect the nano-cutting process, whereas the cooling action of fluid media is as important as the lubrication action on the machining accuracy and surface quality.

Therefore, in this paper, the cutting tool and the workpiece are completely surrounded by the cutting fluid media which not only exist in the action area of tool-chip interface but also exist in the areas of workpiece surface, machined surface, and the rear area of the tool. Hence, the adequate lubrication of fluid media between cutting tool and workpiece can be investigated. Furthermore, the fluid media are set as a constant temperature during nano-cutting process, and the cooling action of the fluid media can also be well studied. Water-based cutting fluid is widely used in ultra-precision machining process, which contains not only water, but also soluble base oil, castor oil, triethanolamine, boric acid, surfactant, polyethylene glycol, and sodium phosphate. Nevertheless, the major constituent of the cutting fluid is water media, and the mass fraction accounts of water in cutting fluid reached about 70%. Due to the difficulties in building MD models of all other substances, and the potential function parameters unknown, the study on nano-cutting with the use of water-based cutting fluid cannot be carried out by molecular dynamic computational simulation methods. Therefore, water medium, which is the main component of cutting fluid, is adopted in this research to replace water-based cutting fluid during nano-cutting process simulation. Based on the established MD model, the nano-cutting process is performed to study the effect of water media on material removal and subsurface defect formation mechanism. The subsurface defect evolution, the variation of cutting force, the temperature distribution of workpiece, and the subsurface defect crystal transformation are investigated by using centro-symmetry parameter (CSP), common neighbor analysis (CNA), and dislocation extract algorithm (DXA) methods.

## Methods

### Simulation Model

**Fig. 1:**
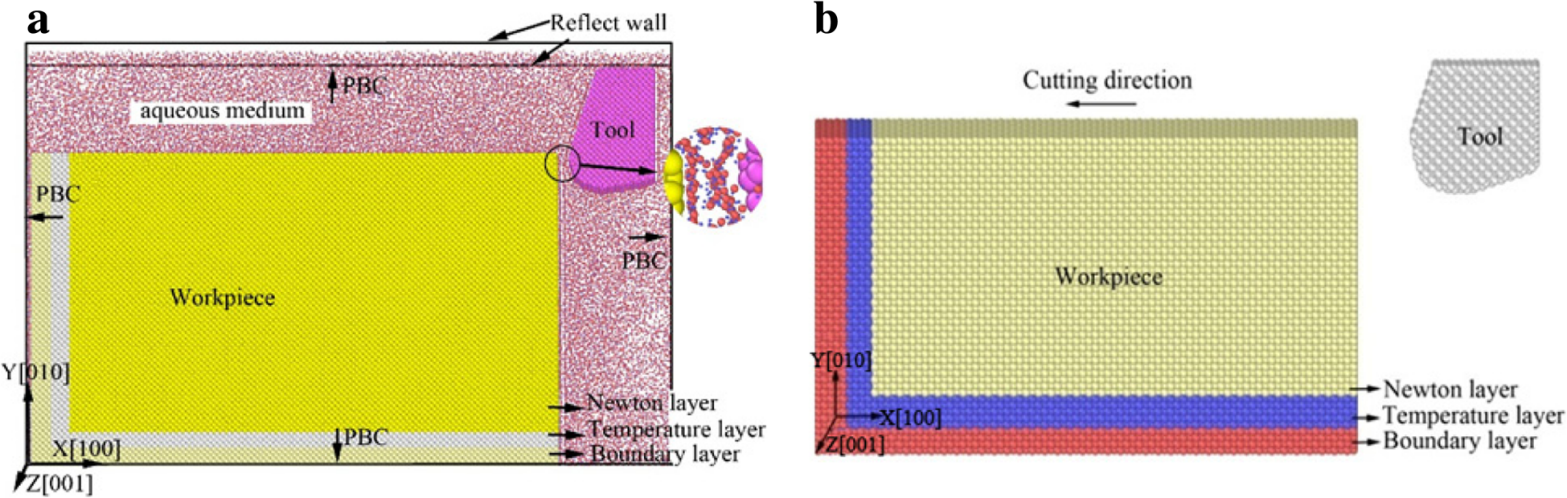
The MD simulation models in nano-cutting. **a** The use of aqueous media. **b** The use of vacuum environment

In order to investigate the effect of fluid media on material removal and subsurface defects evolution in nano-cutting, the MD models with and without aqueous media are established, as shown in Fig. 1. In the models, water molecules are built according to the TIP4P model [23–25]. The CHARMM force field and Lennard-Jones (L-J) potential function are used to precisely calculate the movement of water molecules. It can comprehensively analyze the effect of nonbonding potential energy, bond expansion potential, bending potential energy of bond angle, and the molecular vibration, which makes the simulation of water molecules more accurate. The workpiece material is single-crystal copper, and the machining tool is of diamond materials. It contains 62835 water molecules, 368208 Cu atoms and 2452 C atoms. The workpiece is divided into three parts, which are Newton layer, temperature layer, and boundary layer. In order to reduce the size effect, the periodic boundary condition (PBC) is adopted at [001] and [010]. In order to remain the pressure and density of water media, the reflect walls are used at both sides of the [001] direction. In actual machining process, the coolant takes off most of the cutting heat, so the water environment is set to a constant temperature at 300 K in this work. The MD simulation model with vacuum environment is shown in Fig. 1b, where the initial simulation conditions are similar with the fluids environment model. The different settings between the two models are the relevant settings for aqueous media. The detailed cutting parameters are shown in Table 1.

**Table 1:** MD simulation conditions in 3-D nano-machining

Machining parameters	Value
Potential function	Tersoff, Morse, EAM, Lennard-Jones
Workpiece	Single-crystal copper
Tool	Diamond
Lattice structure	FCC
Workpiece size	30 × 18 × 8 nm
Tool rake angle α	15°
Tool clearance angle β	8°
Tool edge radius *R*	3.0 nm
Cutting direction	(100)[100]
Cutting depth	3 nm
Cutting speed	50 m/s

### Interatomic Potential Functions

In MD simulations, the potential function plays a decisive role in the simulation results. The material properties are fundamentally controlled by the interaction between atoms. In this research, the model is divided into three parts which are workpiece, cutting tool, and aqueous media, and contains four atomic types, which are Cu, C, H, and O atoms. The interactions among different atoms are calculated by Morse potential, embedded-atom method (EAM) potential, Lennard-Jones (L-J) potential, and Tersoff potential. The detailed introduction of the selected potential function between different atoms is shown as Table 2.

**Table 2:** Potential function between different atoms

	Cu	C	H	O
Cu	EAM	Morse	L-J	L-J
C		Tersoff	L-J	L-J
H			L-J	L-J
O				L-J

### Morse Potential

The interaction between Cu atoms in the workpiece and C atoms in the cutting tool is calculated by Morse potential which is shown as Eq. (1) [26].

1 $$ u\left({r}_{ij}\right)=D\left[\exp \left(-2\alpha \left({r}_{ij}-{r}_0\right)\right)-2\exp \left(-\alpha \left({r}_{ij}-{r}_0\right)\right)\right] $$

where *r*_0_, *α*, and *D*, respectively, are atomic spacing, elasticity modulus, and binding energy. The values of *r*_0_, *α*, and *D* are shown in Table 3.

**Table 3:** Parameters value in Morse potential

*r*_0_ (Ả)	*α* (Ả^−1^)	*D*(eV)
2.05	5.140	0.087

### EAM Potential

The interatomic function among Cu atoms in the workpiece is described by EAM potential which is shown as Eqs. (2) and (3) [27, 28].

2 $$ E\kern0.5em =\kern0.5em \sum \limits_i^N\left[F\left({\rho}_i\right)\kern0.5em +\kern0.5em \sum \limits_{j\kern0.5em >\kern0.5em i}^Nu\left({r}_{ij}\right)\right] $$

3 $$ {\rho}_i\kern0.5em =\kern0.5em \sum \limits_jf\left({r}_{ij}\right) $$

### Lennard-Jones Potential

Lennard-Jones potential function is a dual potential, which includes the interaction of both long-range Coulomb force and short-range van der Waals force between atoms. The L-J potential is often used to simulate the liquid materials. In this paper, the Lennard-Jones potential is used to calculate the interaction between water molecules and other atoms, which is shown as Eq. (4) [29].

4 $$ {U}_{L-J}(r)\kern0.5em =\kern0.5em 4\varepsilon \left[{\left(\frac{\sigma }{r}\right)}^{12}\kern0.5em -\kern0.5em {\left(\frac{\sigma }{r}\right)}^6\right] $$

where *σ* is the equilibrium separation when the interaction potential energy is equal to zero and *ε* is the depth of potential energy trap.

For different materials, *σ* and *ε* can be calculated by Eqs. (5) and (6) [29].

5 $$ {\sigma}_{\alpha \beta}\kern0.5em =\kern0.5em \frac{\sigma_{\alpha \alpha}\kern0.5em +\kern0.5em {\sigma}_{\beta \beta}}{2} $$

6 $$ {\varepsilon}_{\alpha \beta}\kern0.5em =\kern0.5em \sqrt{\varepsilon_{\alpha \alpha}\cdot {\varepsilon}_{\beta \beta}} $$

The interatomic L-J potential parameters which are used in this research are listed in Table 4.

**Table 4:** Interatomic L-J potential parameters

	Cu-O	Cu-H	C-O	C-H	O-O	O-H	H-H
$\sigma \Big(\overset{\circ }{\mathrm{A}\Big)}$	2.7475	1.3653	3.6	3.0	3.16435	0	0.40
ε(eV)	0.038	0.02021	0.0075	0.002385	0.00706	0	0.00199

### Terseff Potential

The interaction between carbon atoms in the diamond tool is calculated by Tersoff potential which is shown as Eqs. (7) and (8) [30].

7 $$ E\kern0.5em =\kern0.5em \frac{1}{2}\sum \limits_{i\ne j}{V}_{ij} $$

8 $$ {V}_{ij}\kern0.5em =\kern0.5em {f}_c\left({r}_{ij}\right)\left[{V}_R^{\hbox{'}}\left({r}_{ij}\right)\kern0.5em +\kern0.5em {b}_{ij}{V}_A\left({r}_{ij}\right)\right] $$

where *f*_*c*_(*r*_*ij*_) is the truncation function between atoms, *V*_*A*_(*r*_*ij*_) is the dual potential of absorption term, *V*_*R*_(*r*_*ij*_) is the dual potential of repulsion term, and *r*_*ij*_ is atomic distance between atom *i* and atom *j*.

### Defect Analysis Methods

In nano-cutting of single-crystal copper, deformation and dislocations are nucleated at the subsurface of the workpiece. In this paper, the centro-symmetry parameter (CSP) is introduced to analyze the dislocation nucleation and defect evolution of the workpiece. For face center cubic (FCC) materials, the CSP value can be calculated by Eq. (9) [31].

9 $$ CSP\kern0.5em =\kern0.5em \sum \limits_{i\kern0.5em =\kern0.5em 1}^6{\left|{R}_i\kern0.5em +\kern0.5em {R}_{i+6}\right|}^2 $$

where *R*_*i*_ is the neighboring atoms with the same distance and *R*_*i + 6*_ is the neighboring atoms with an opposite direction. The CSP values of FCC crystal, partial dislocation, stacking faults, and surface atoms are 0, 2.1, 8.3, and 24.9, respectively [32]. The range of CSP value for typical crystal structure and atomic coloring are shown as Table 5.

**Table 5:** The range of CSP values for typical crystal structure

Crystal structure	Range of CSP value	Atomic coloring
Ideal FCC	CSP≤3	Default
Stacking fault	3<CSP≤7	Red
Partial dislocation	7<CSP≤9	Orange
Surface atoms	9<CSP≤20	Yellow
Surface defect atoms	CSP>20	Green

The CSP method is able to identify the atomic configuration, but cannot recognize the local atomic crystal structure state of the workpiece. Therefore, the common neighbor analysis (CNA) is introduced to identify the local crystal structure of the workpiece. In the original CNA method proposed by Honeycutt [33], the various structures are represented by diagrams. Currently, it is improved to rapidly identify five kinds of structures in the OVITO software [34, 35], which, respectively, are face center cubic (FCC), close-packed hexagonal (HCP), body centered cubic (BCC), icosohedral (ICO), and unknown. In this paper, dislocation extract algorithm (DXA) [36] is also introduced to analyze the evolution of dislocation defect. By DXA method, the different crystal structures in the workpiece will be marked with different colors and the dislocation defects in the workpiece will be represented by lines of different colors.

## Results and Discussion

### Subsurface Defect Evolution in Nano-Cutting Process with Aqueous Media

**Fig. 2:**
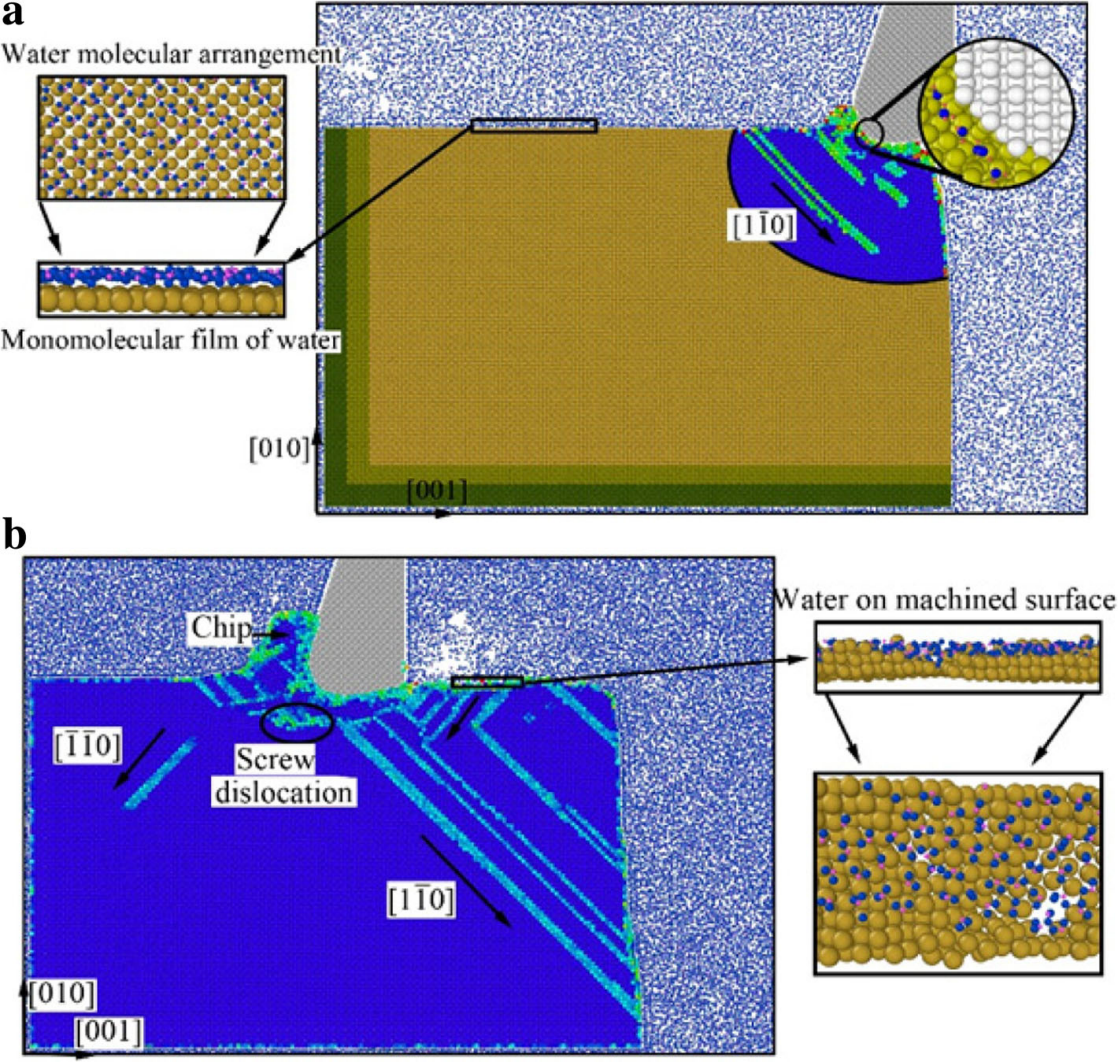
Sectional view of cutting system with aqueous media in nano-cutting process (color online). **a** Cutting distance l = 5nm. **b** Cutting distance l = 15nm

The sectional view of cutting system is shown in Fig. 2, which contains the cutting tool, workpiece, and water media during nano-cutting process. In order to clearly perceive the plastic deformation of the workpiece, the CSP method is used to analyze the results. The snapshots are colored by the value of CSP partially as shown in Fig. 2, in which Fig. 2a is at 5 nm of cutting distance and Fig. 2 b is at 15 nm of cutting distance. It can be seen that a layer of compact water film is formed on the surface of single-crystal copper which is shown as “monomolecular film of water” in Fig. 2a. The water film spread all over the surfaces of the cutting tool and workpiece, in which the oxygen atoms occupy the center of the single-crystal copper lattice. The regular arrangement of water molecules is the result of the combined action of long-range Coulomb force and van der Waals force between water molecules and copper atoms.

In nano-cutting process, the slip deformation is resulted by the early compressional shearing action of cutting tool and is stored in the formed crystal lattice as strain energy. When the strain energy accumulation reached a certain level, the strain energy is released. And then, the lattice of single-crystal copper is rearranged, which caused dislocation nucleation and extension along the $\left[1\overline{1}0\right]$ direction, as shown in Fig. 2a. It can be shown from Fig. 2a that the monomolecular film of water is formed on the workpiece surface. Furthermore, the water molecules are penetrated into the subsurface of the workpiece in the action area of the tool-chip interface, which is shown as the top right illustration in Fig. 2a. Due to the lubrication action of water molecules that existed between the cutting tool and the workpiece, the compressional shearing action of cutting tool is attenuated. And the strain energy accumulation decrease and the dislocations extend insufficiently. In addition, the aqueous media took away a lot of cutting heat, and the energy of nucleated dislocation expansion is insufficient. Therefore, the dislocation extension is insufficiency and the dislocation line in subsurface of workpiece is inconspicuous, as shown in Fig. 2a.

As the cutting tool is moving forward, the workpiece suffered the extrusion and friction action generated by the flank face of cutting tool. Under the extrusion and rubbing action of the cutting tool, a large number of dislocations nucleated and extended in the subsurface of workpiece. One part of these dislocations moves upward along the rake face and ultimately is removed as a cutting chip, as shown in Fig. 2b. Another part of these dislocations moves downward along the rake face of the cutting tool and is transformed to roughness machined surface after the extrusion friction action of the flank face of cutting tool, which is shown in Fig. 2b as “water arrangement on machined surface.” Other parts of the dislocations move inward along the $\left[\overline{1}\overline{1}0\right]$ and $\left[1\overline{1}0\right]$ slip plane and disappear inside the workpiece, which results in the formation of screw dislocation, as shown in Fig. 2b. Due to the chip height increasing gradually, the water molecules in front of the chip cannot flow over the chip to behind the cutting tool in the later period of cutting process. And the density and pressure of water media behind the cutting tool decrease rapidly, which results in the cutting heat being taken away untimely during nano-cutting process. Therefore, the nucleated dislocations have enough energy to extend inside of the workpiece, as shown in Fig. 2b.

**Fig. 3:**
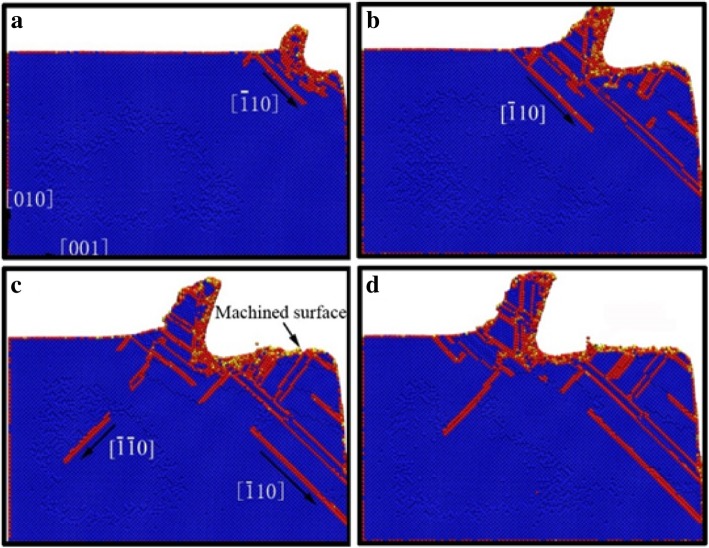
Subsurface defects distribution of workpiece with aqueous media in nano-cutting process. The cutting distances of **a**, **b**, **c**, and **d** are 5 nm, 8 nm, 12 nm, and 15 nm, respectively.

**Fig. 4:**
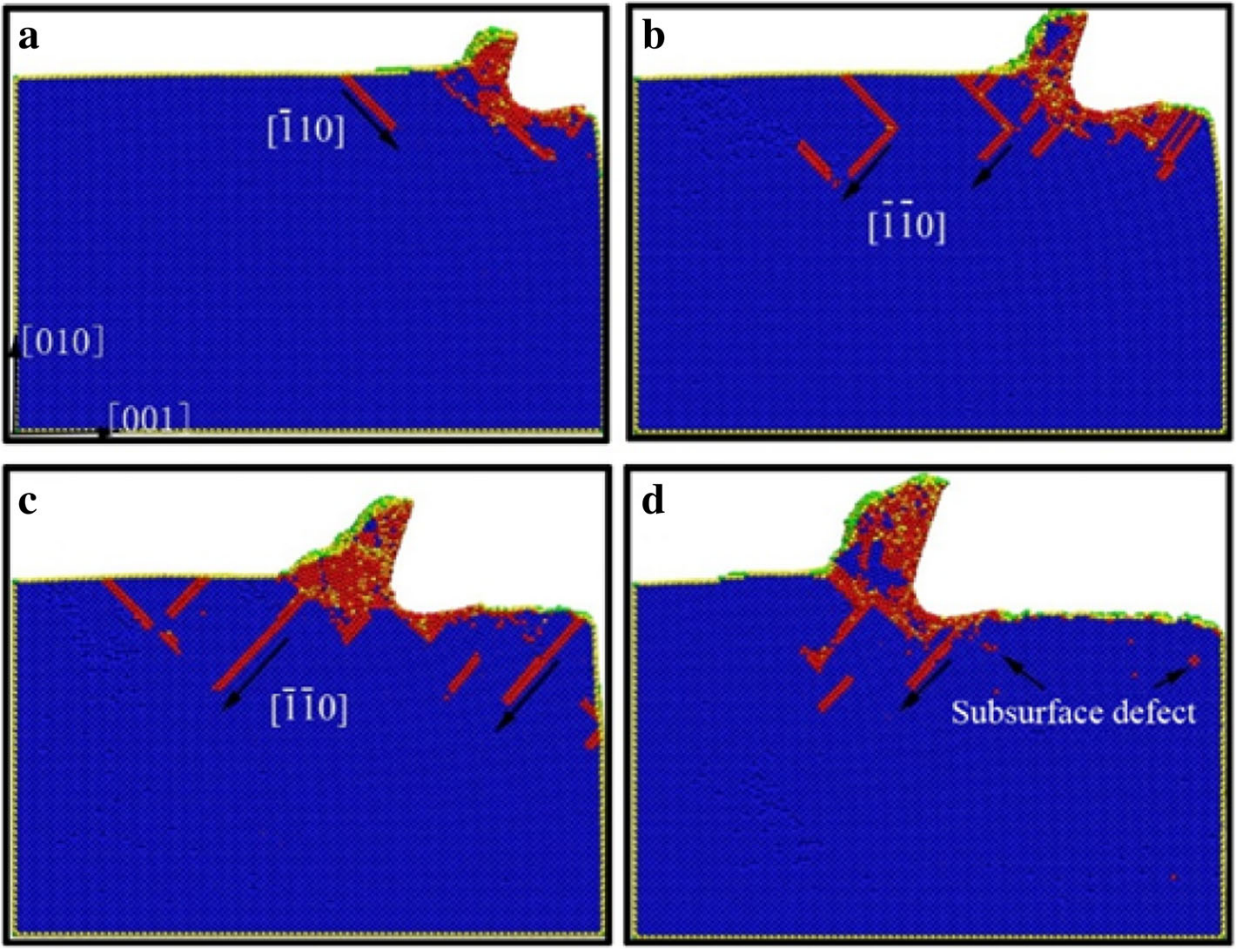
Subsurface defects distribution of workpiece during nano-cutting process in vacuum environment. The cutting distances of **a**, **b**, **c**, and **d** are 5 nm, 8 nm, 12 nm, and 15 nm, respectively.

**Fig. 5:**
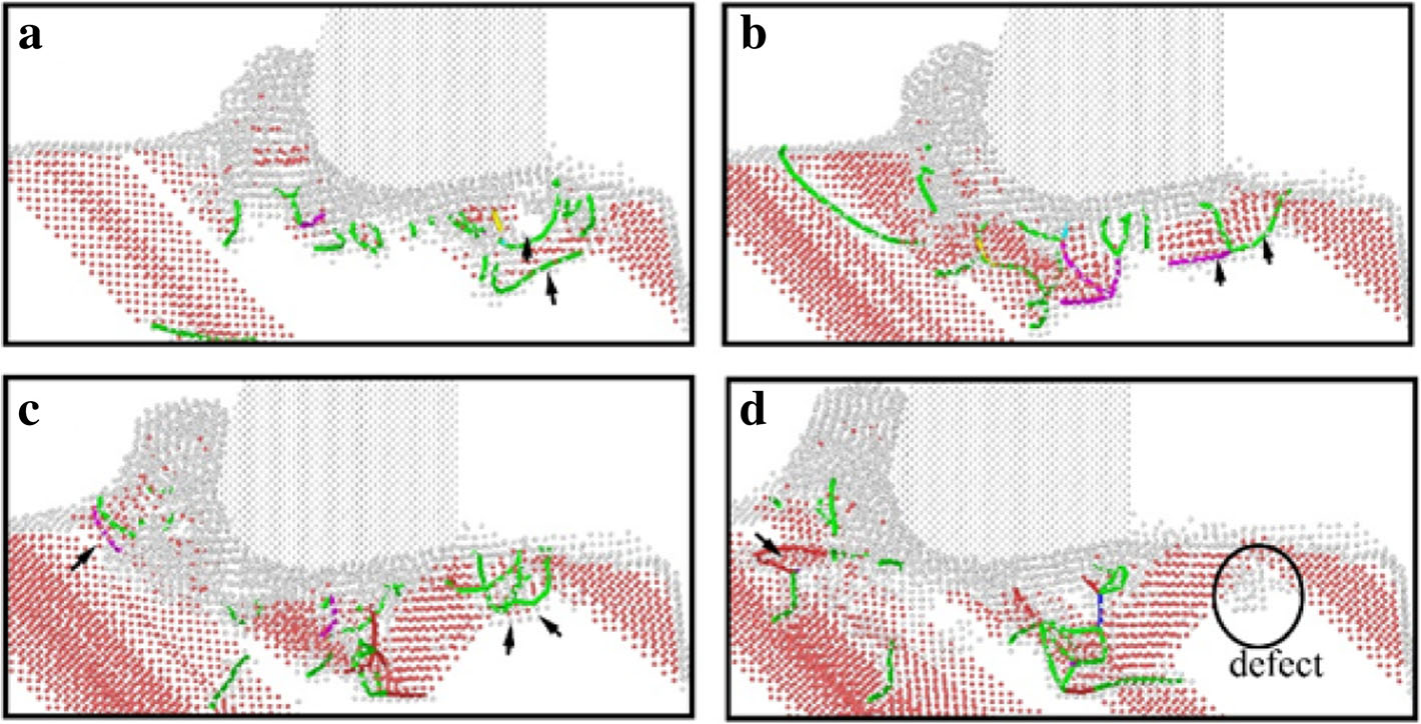
The DXA image of workpiece in the early stage of nano-cutting. Dislocations are colored based on following scheme: deep blue for perfect dislocation, green for Shockley dislocations, pink for Stair-rod dislocation, yellow for Hirth dislocations, light blue for Frank dislocations, and red for unidentified dislocations. The cutting distances of **a**, **b**, **c**, and **d** are 7 nm, 8 nm, 9 nm, and 10 nm, respectively

**Fig. 6:**
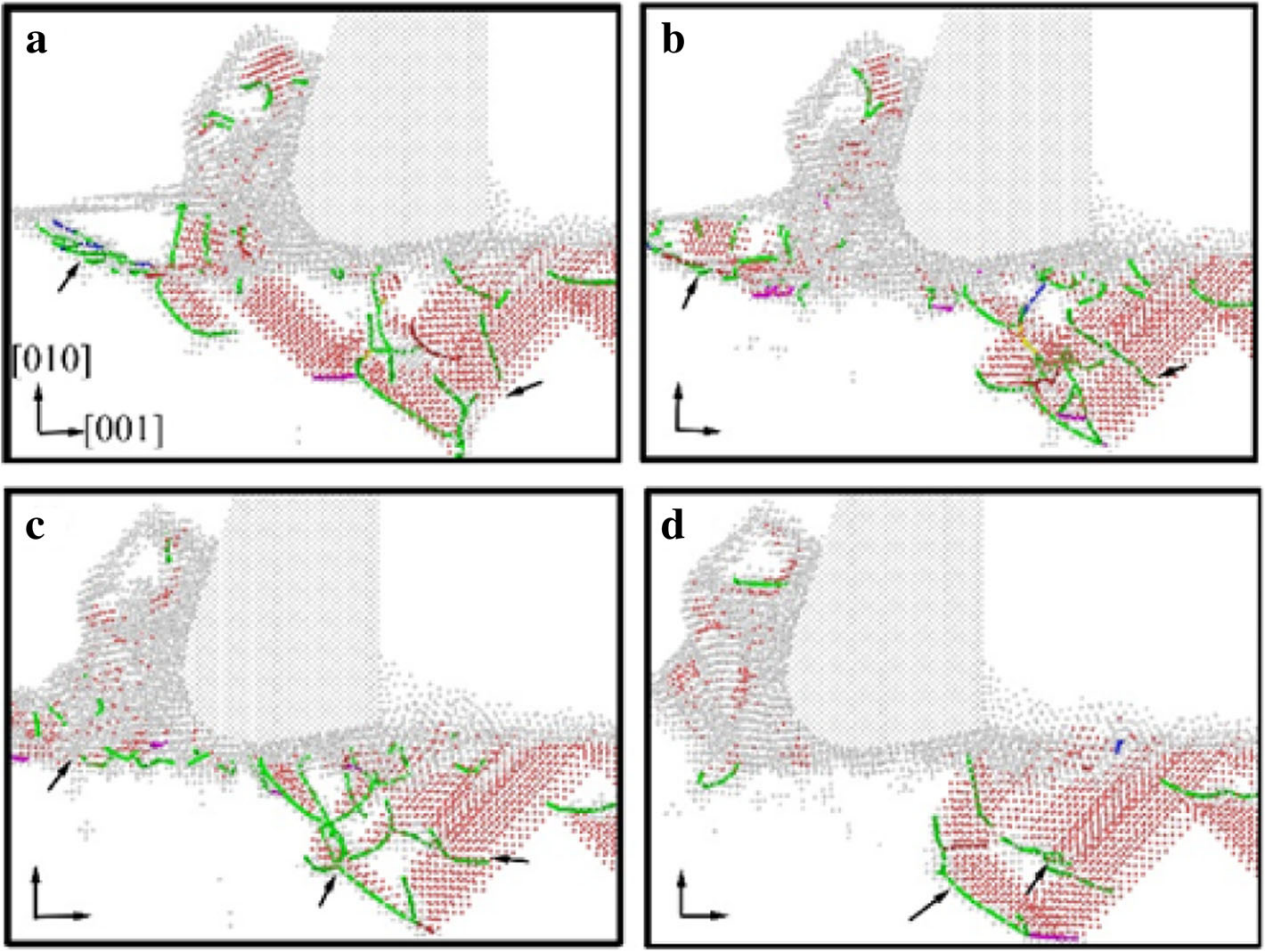
The DXA image of workpiece in the later period of nano-cutting. The coloring scheme of dislocations is the same as Fig. 5.The cutting distances of **a**, **b**, **c**, and **d** are 17 nm, 18 nm, 19 nm, and 20 nm, respectively

In order to elucidate the underlying deformation of dislocations and local atomic crystal structure of single-crystal copper during nano-cutting process, the CSP and DXA analysis methods are introduced. The analysis results are exhibited as shown in Figs. 3, 4, 5, and 6, among which Figs. 3 and 4 are rendered according to the CSP value and Figs. 5 and 6 are colored by DXA analysis result.

The subsurface defect distribution of the workpiece in nano-cutting process with aqueous media is shown in Fig. 3, in which the water media is not display to observe the dislocation defect evolution more clearly. The yellow, green, red, and orange areas represent surface atoms, surface defect atoms, dislocation atoms, and subsurface defect atoms, respectively. The dislocation distribution and extension of the workpiece in nano-cutting process without the aqueous media is shown as Fig. 4. It can be seen from the two figures that the nucleated dislocations migrate along the $\left[\overline{1}10\right]$ slip vector during nano-cutting process with aqueous media, but extend along the $\left[\overline{1}\overline{1}0\right]$ slip vector in nano-cutting under vacuum media. As we known, the shear action of cutting tool makes dislocations extending along the direction against the tool forward, which is $\left[\overline{1}10\right]$ slip vector. The friction action of cutting tool leads to the dislocation migrating along the direction orthokinetic tool movement, which is a $\left[\overline{1}\overline{1}0\right]$ slip vector. During nano-cutting process in vacuum environment, the actions of cutting tool on workpiece are shearing action of rake face and friction action of flank face, while the formation of machined surface and subsurface defects is triggered by the friction action of flank face. Therefore, the dislocation extension spread along $\left[\overline{1}\overline{1}0\right]$ slip vector during nano-cutting process in vacuum. Due to the lubrication of water molecules existing between tool and workpiece, the friction action of cutting tool is reduced. Thus, the shearing action plays an important role in the formation of machined surface and subsurface defects. Therefore, the dislocations mainly extend along the $\left[\overline{1}10\right]$ slip vector in nano-cutting with aqueous media.

From Figs. 3 and 4, it can be found that the scale of subsurface defects in water media is greater than in vacuum during nano-cutting process. Actually, the dislocation defects spread all over the machined area and extend deep inside the workpiece. The cutting heat is taken away by water media, and the energy of defect atomic is decreased. Hence, the subsurface defects do not have enough energy to be annihilated. Therefore, the dislocation defect residue is increased. The depth of subsurface defect layer is relatively higher for the nano-cutting process with water media. Due to the interactions among water molecules, carbon atoms, and copper atoms, the extrusion friction between cutting tool and workpiece is attenuated and the atomic disorder of machined surface is aggravated in the formation process of machined surface. Furthermore, the defect residue in subsurface is exacerbated and the subsurface residual stress is increased.

In order to better reveal the effect of water media on the dislocation defect evolution process, the DXA method is used to analyze the workpiece in nano-cutting process with aqueous media, in which the early stage and the late period are shown as Fig. 5 and 6, respectively. A stable crystal defect is found existing in several layers atoms below the machined surface in the early stage of nano-cutting process, which is located between two stacking faults, as shown in Fig. 5d. The existence of the crystal defects will affect the quality of machined surface and even lead to the generation of micro crack on the machined surface. Therefore, the formation of the defect evolution process is studied. It can be seen from Fig. 5a that a lot of Shockley partial dislocations is nucleated under the friction action of the flank face of cutting tool in the early instant of the defect formation. These Shockley dislocations are evolved into a V-shaped dislocation loop during the cutting tool going forward, as shown in Fig. 5b. Subsequently, the V-shaped dislocation is gradually evolved into serial Shockley partial dislocations. Finally, the partial dislocations are transformed into residual defect in subsurface. Due to the cutting heat being taken away by water media, the defective atoms have too little energy to be annihilated and are transformed into immobile defect underlying the machined surface. The roughness of machined surface will be increased, and residual stress in subsurface will cause more aggravation. Furthermore, a surface microcrack can be induced by the defect.

The effect of water media on the shearing slip action of cutting tool during chip removal process is investigated by DXA method, which is shown as Fig. 6. It can be seen from Fig. 6a that a large number of Shockley partial dislocations are nucleated in front of cutting tool. And the shear-slip plane is constituted by these dislocations. In the following cutting process, serial stacking faults and partial dislocations are nucleated and are extended on the shear-slip plane. Under the nucleation and motion of the dislocations in front of the cutting tool, the cutting chip is removed gradually along the shear-slip plane, as shown in Fig. 6c. Meanwhile, the extrusion friction effect of the tool rake face is abated because of the lubrication action of water media. The dislocation nucleation and propagation is insufficient and the subsurface defect residual is unobvious during nano-cutting process, as shown in Fig. 6a–d. Correspondingly, the shearing action of the cutting tool on the workpiece becomes more significant. Therefore, the formed chip is more easily to be removed under the participation of water media in nano-cutting process. And the main cutting force will decrease simultaneously, which will be discussed in detailed later in this paper.

### Effect of Water Media on the Variation of Cutting Force and Cutting Heat

In the cutting process, the material removal is realized by the extruding and shearing action of the cutting tool. Due to the strength, stiffness, and toughness of metal material, the rake surface of the cutting tool suffered the reaction force generated by the resistance to deformation of the workpiece material during the material removal process. And the flank surface of the tool is affected by the friction force resistance of the machined surface. These forces composed the cutting force together. Synchronously, the applied work by the shear deformation of cutting chips as well as the rubbing action between cutting tool and workpiece is converted into cutting heat which resulted in the increased temperature of workpiece. Accompanying with the accumulation and release of material strain energy, dislocation nucleation and expansion occurred at the subsurface layer of the workpiece, which induced the fluctuation of cutting force and cutting heat with the cutting distance.

**Fig. 7:**
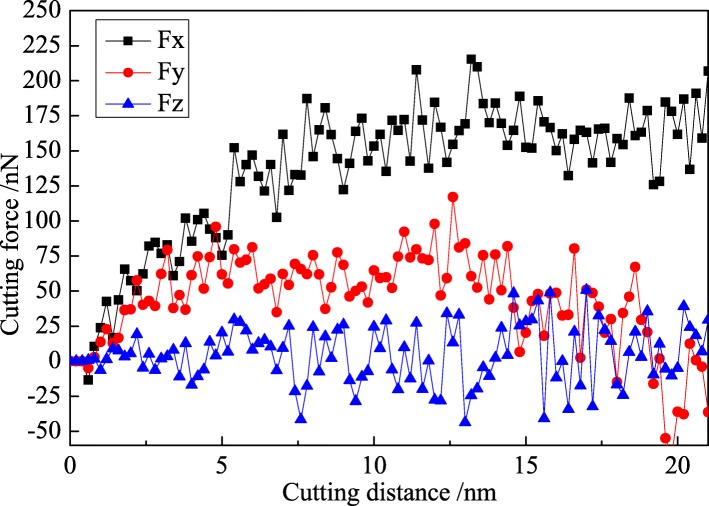
Variation curve of cutting force in nano-cutting with vacuum environment

**Fig. 8:**
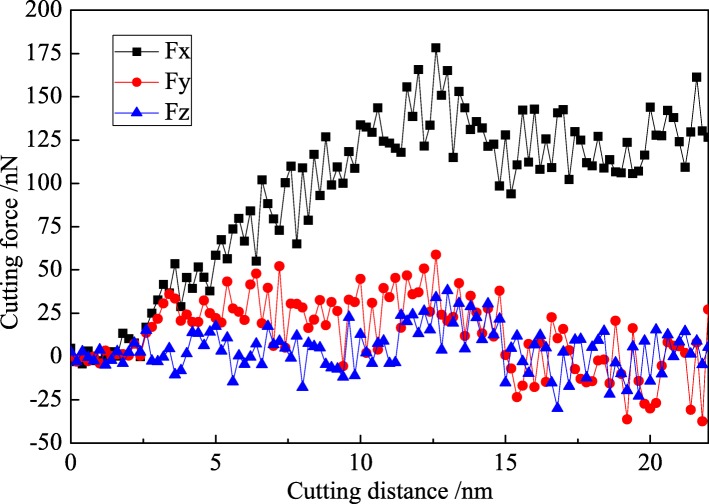
Variation curve of cutting force in nano-cutting with water media

In this research, the participation of water media makes great influence on the variation of cutting force and cutting heat. The variation curves of cutting force with cutting distance in nano-cutting process with and without the use of aqueous media are shown as Figs. 7 and 8, in which the black, red, and blue curve, respectively, are feed force (Fx), back force (Fy), and tangential force (Fz). Due to the PBC being adopted at the Z direction and the diamond tool is columnar along the Z direction in the simulation, the average tangential force (Fz) is at the level of 0 nN in both Figs. 7 and 8.

The cutting process is divided into the initial cutting stage and stable cutting stage as shown in Fig. 7. In the initial cutting stage, the feed force and back force are increased sharply in a straight line. And they reached their maximum values when the cutting tool cut into the workpiece completely. In the stable cutting stage, the cutting forces fluctuate up and down in their equilibrium position, and the feed force reaches more than 200 nN and the mean cutting force is about 180 nN. The back force is very small and decreases gradually in the late stable cutting stage. The maximum value of the bake force is under 100 nN, and the average value is around 50 nN. Generally speaking, the specific value between average back force and average feed force (Fy/Fx) represents the friction coefficient between tool material and workpiece material in machining process. In this research, the friction coefficient between diamond and monocrystal copper is 0.278 under vacuum environment.

The feed force and the back force are decreased in nano-cutting with water media compared with vacuum environment, as shown in Fig. 8. The maximum feed force reaches 150 nN, and the mean feed force fluctuates at 120 nN. The variation tendency of back force is similar with the back force in vacuum environment, and the mean force is at about 25 nN. In nano-cutting process with the use of water medium, the friction coefficient between the cutting tool and the workpiece is reduced due to the lubrication of water. And then the frictional resistance suffered by the flank surface of cutting tool is reduced, which effectively enhances the extrusion shearing action of the rake surface of cutting tool. The removal of the workpiece material is easier to be removed. Hence, the cutting force is reduced. It can be seen from the foregoing analysis that the water molecules acted as a lubricant to prevent the friction between the cutting tool and the workpiece. Therefore, the values of feed force and back force are reduced in the water media. The specific value between the feed force and the back force is 0.208. In another words, the frictional coefficient between diamond and copper in water media is 0.208, much fewer than they are in vacuum environment (0.278).

Compared with the fluctuation of the Fy in Figs. 7 and 8, it is indicated that the Fy component decreases considerably after 15 nm of cutting distance in both cases with vacuum and water media while the Fx value is almost stable until 20 nm of cutting distance. The dynamic balance between dislocation nucleation and annihilation is achieved, and the chip is removed steadily in nano-cutting process, which results in the cutting force almost stable with the cutting distance before 20 nm. When the cutting process is carried out at a certain distance (15 nm in this research), the dynamic equilibrium between the new dislocation nucleation and the previous dislocation annihilation is established. And the scale of the internal defects of the workpiece is stabilized at a certain level. The dislocation nucleation and annihilation applied an effect on cutting tool along +Y direction, which leads to the Fy component decrease. Besides, the stable cutting chip is removed after 15 nm of cutting distance, and the applied force on the cutting tool from the chip is decreased along the Y direction. Thereby, the value of Fy is reduced. However, when the cutting distance is greater than 15 nm till 20 nm, the emotion of dislocation defect and the removal of chip cannot bring a different influence on the cutting tool along the X direction. Therefore, the value of main cutting force (Fx) is almost stable.

**Fig. 9:**
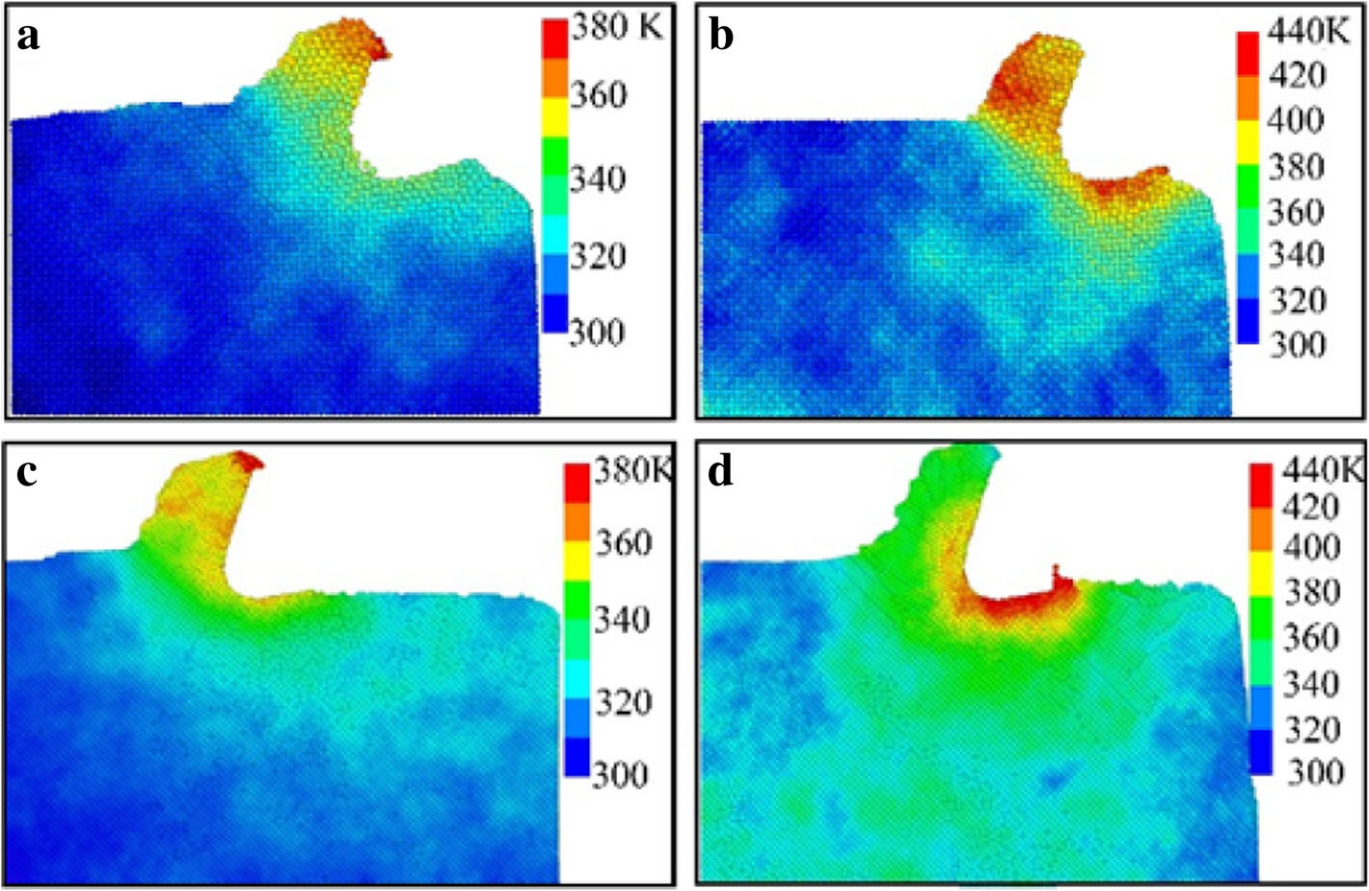
Temperature distribution of workpiece in nano-cutting. **a** and **c** are in water media, **b** and **d** are in vacuum environment

The temperature distribution of the workpiece during the nano-cutting process with and without the use of aqueous media is shown as Fig. 9. The cutting distances of Figs. 9a and b and Figs. 9c and d are 5 nm and 12 nm, respectively. It can be seen from Figs. 9b and d that the temperature of workpiece is distributed as a concentric gradient. The highest temperature is spread all over shear-slip zone and friction zone of the workpiece in a vacuum environment, which is above 420 K. The temperature of the chip and machined surface is higher than other regions, which is ranged from 360 to 390 K. For the whole workpiece, the temperature is at a high level, which is ranged from 340 to 360 K. From Figs. 9a and c, the temperature of the workpiece is also distributed as a concentric gradient and the highest temperature is distributed at the top area of the chip, which is around 370 K. The temperature value of the whole workpiece is at a lower level which is lower than 320 K. The temperature of the shear-slip area, the friction zone, and the machined surface are higher than other areas, which is ranged from 320 to 340 K.

The highest temperature area of workpiece is transferred from the friction area to the cutting chip during nano-cutting process with water media as shown in Fig 9. And the highest and whole temperature of the workpiece are significantly reduced with the additional use of water media, and the temperature drop reached about 40–60 K. Due to the lubrication effect of water molecules, the friction between cutting tool and machined surface is reduced during nano-cutting process with water media. Hence, the temperature of the friction area declined dramatically. Meanwhile, the maximum shearing deformation occurred at the chip area and the maximum lattice deformation energy is stored in the chip, which makes the temperature of the chip higher than the friction area. Therefore, the highest temperature area is transferred from friction area to the cutting chip. Synchronously, a large amount of cutting heat is taken away by the water media which play a role in cooling the tool, workpiece, and cutting area. And the thermal movement of the monocrystal copper molecules is weakened. Furthermore, the kinetic energy of atomic thermal motion and the lattice deformation energy are decreased significantly. Therefore, the overall temperature and the highest temperature of the workpiece is reduced, whose degree of reduction arrived at 40–60 K. Finally, the thermal stress and thermal deformation of the workpiece are significantly reduced. Because of the participation of water media, the friction action between the flank surface of cutting tool and workpiece is weakened in cutting process. Then, the generation of heat by friction between cutting tool and workpiece is reduced. Thereby, the highest temperature area of the workpiece is transferred from the friction area of flank surface to the chip area. More importantly, the cooling effect and lubrication of water media will affect the nucleation, expansion, and annihilation of the dislocation in subsurface of the workpiece and ultimately affect the formation and evolution of the subsurface damage layers of the workpiece.

### Effect of Aqueous Media on Subsurface Defects Structural Transformation

**Fig. 10:**
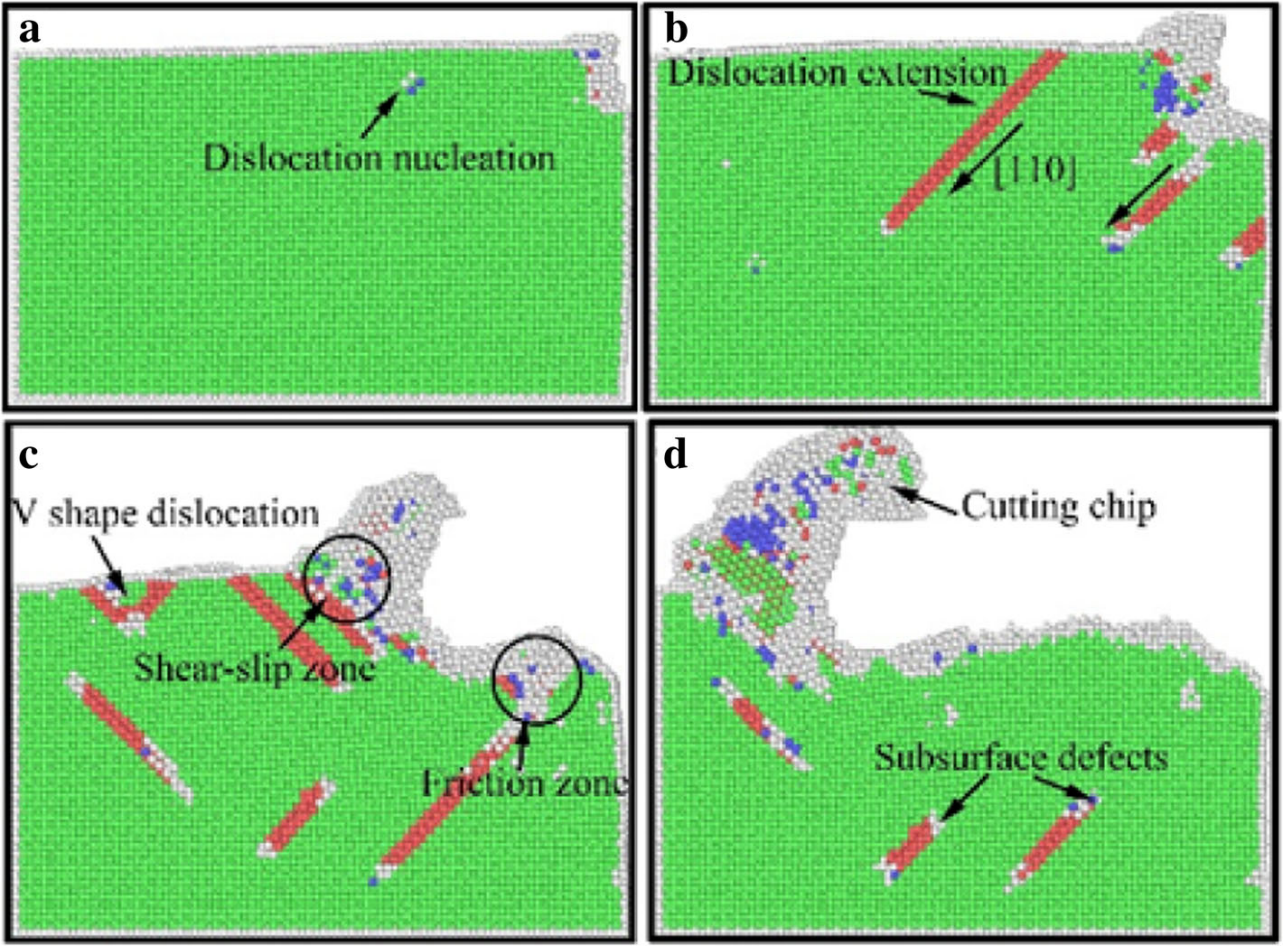
Subsurface defect evolution of workpiece in nano-cutting without aqueous media. The green, red, blue, and grey area are representative of FCC, HCP, BCC, and unknown structure. The cutting distances of a, b, c, and d are 1 nm, 3 nm, 8 nm, and 15 nm, respectively

**Fig. 11:**
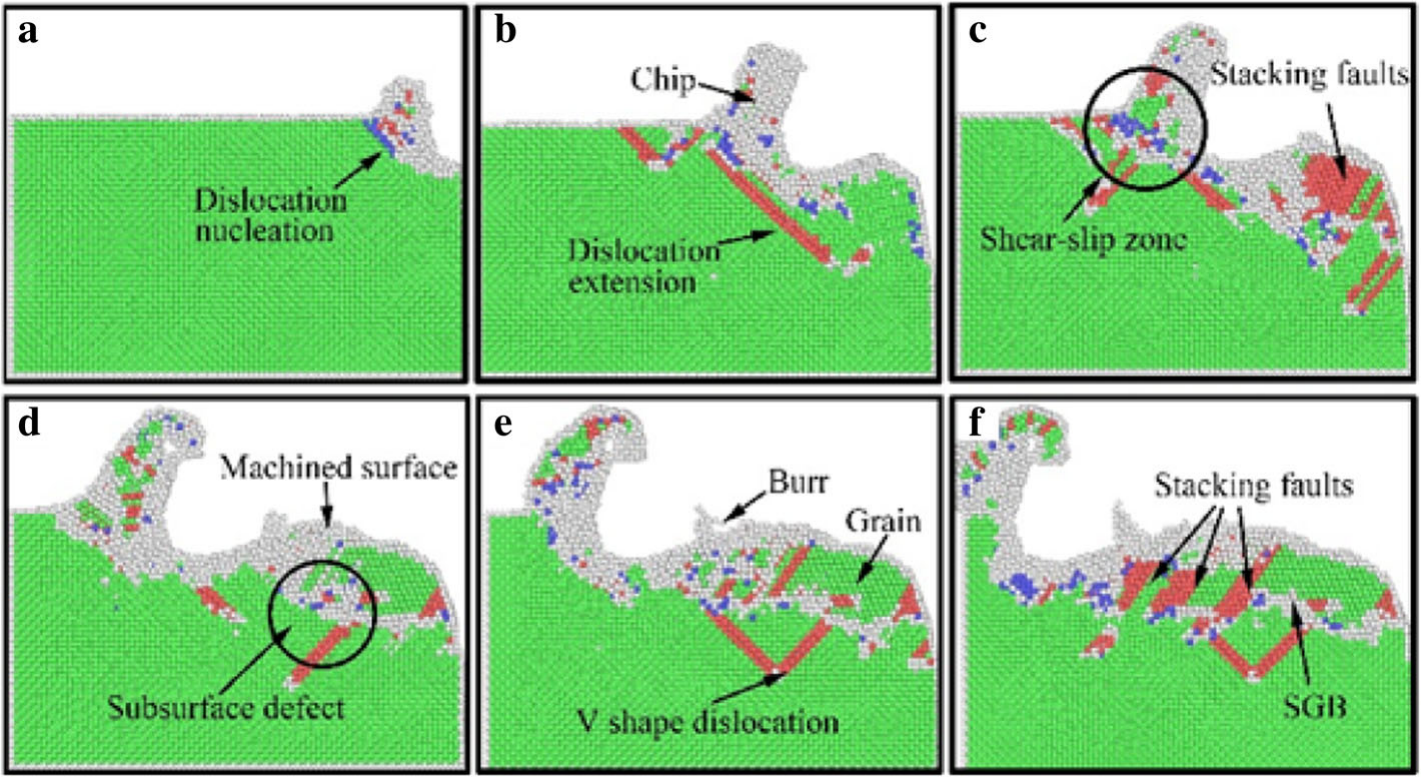
Surface and subsurface defect distribution in nano-cutting for workpiece with water media. The green, red, blue, and grey area are representative of FCC, HCP, BCC, and unknown structure. The cutting distances of a, b, c, d, e, and f are 3 nm, 8 nm, 12 nm, 15 nm, 18nm, and 20nm respectively

In order to clearly identify the subsurface defects of the workpiece in nano-cutting, the CNA method is used to analyze the workpiece after nano-cutting. The workpiece is colored by different atomic structure. The defect structural distribution of the workpiece during nano-cutting process with and without the use of aqueous media is shown as Figs. 10 and 11, in which the green, red, blue, and grey are FCC, HCP, BCC, and unknown structure, respectively.

In nano-cutting process under vacuum environment, dislocation nucleation occurred at the subsurface of workpiece under the extrusion and shearing action of the cutting tool, and the crystal structure is transformed into BCC, which is shown as Fig. 10a. The nucleated dislocation is extended along $\left[\overline{1}\overline{1}0\right]$ direction, and the crystal structure is transformed into HCP. The crystal structure of many atoms in the shear-slip region become BCC, as shown in Fig. 10b. Two partial dislocations are extended along the $\left[\overline{1}\overline{1}0\right]$ and $\left[1\overline{1}0\right]$ directions, hindered each other, and be composed of Lomer-Cottrell dislocation lock. Finally, a typical V-shaped dislocation loop is formed, as shown in Fig. 10c. Part of the atoms in the shear-slip zone are moved upward along the rake face and are removed as cutting chip. The other part of the atoms are migrated downward along the flank face and are formed into the roughness machined surface by the extrusion and friction of the cutting tool, as shown in Fig. 10d.

The subsurface defect distribution and evolution of workpiece in nano-cutting with the use of water media is shown as Fig. 11. It can be seen from the figure that the mechanisms of dislocation nucleation and crystal structure transformation are similar with the cutting process in vacuum environment. The main difference is that the dislocation nucleation and expansion is insufficient in the nano-cutting process of water media. Besides, there are many stacking faults nucleated in the subsurface of the workpiece. The structure of stacking faults is transformed into HCP structure. Nevertheless, a typical defect “similar-to-grain boundary (SGB)” is formed in the subsurface of the workpiece.

In nano-cutting process, under the action of extrusion, shearing and friction by cutting tool, intense deformation of the workpiece is generated. Plenty of deformation energy and cutting heat are produced. The atomic lattice reconfiguration of subsurface is produced by the release of cutting heat and strain energy. And the subsurface defects and local crystal structure transformation are formed, as shown in Fig. 11a and 11b. When the water media participated in the nano-cutting process, most of the heat and energy is taken away. Hence, the dislocation defects have inadequate energy to extension and movement. Furthermore, the stacking faults are annihilated in the subsurface of the workpiece where the crystal defect structure stayed behind, as shown in Fig. 11c, whereafter these crystal defect structures are connected as a whole and are composed of the subsurface damage (SSD) layer together with the newly formed dislocations, as shown in Fig. 11d. After the following MD relaxation, some subsurface dislocation defects are disappeared and transformed into FCC structure, and the structure similar to “grain” is formed between machined surface and subsurface defects layer, as shown in Fig. 11e, while the original subsurface defects are transformed into a typical structure “similar-to-grain boundary (SGB),” as shown in Fig. 11f. On the SGB structure, a typical V shape dislocation loop is formed, as shown in Fig. 11e, f.

The metamorphic layer is obviously formed by the influence of the formation of SGB and “grain” structure in the subsurface of workpiece. Moreover, the new formed crystal structures which are similar with polycrystalline material can influence the mechanical performance and processability of single-crystal materials. Besides, it will affect even the performance of machined nano-components.

## Conclusions

Based on the established MD models of single-crystal copper with and without the use of aqueous media, the simulation of nano-cutting process is carried out. The effects of fluid media on material removal and subsurface defect evolution are analyzed. The subsurface defect evolution, variation of the cutting force, the temperature distribution, and the subsurface defects crystal structure transformation of the workpiece are investigated by using CSP, DXA, and CNA methods. The novel results can be summarized as follows.

(1) The material removal of workpiece is realized by the shearing extrusion action of cutting tool on workpiece; the participation of water media has no effect on the mechanism of materials removal. Due to the lubrication action of water molecules existing between the cutting tool and the workpiece, the deformation of workpiece is decreased, the cutting force is reduced, and the height of cutting chip and depth of subsurface damage layer are lowered.

(2) The highest temperature area is transferred from the friction area to the cutting chip during nano-cutting process with the additional use of water media. And the highest and whole temperature of the workpiece are significantly reduced, and the temperature drop reached about 40–60 K. Thereby, the thermal deformation of the workpiece is reduced and the amount of subsurface defect atoms is decreased.

(3) In the subsurface layer of the workpiece, the crystal structures of nucleated dislocations are transformed into BCC, and the extended dislocations are transformed into HCP. The atomic crystal structures in the shear-slip region are becoming BCC. Under the effect of fluid media, the subsurface defects are transformed into a typical defect structure “similar-to-grain boundary (SGB)” in SSD layer, which can influence the mechanical performance and processability of single-crystal materials. Besides, it will affect even the performance of the machined nano-components.

## Abbreviations

MD: Molecular dynamics
CSP: Centro-symmetry parameter
CNA: Common neighbor analysis
DXA: Dislocation extract algorithm
PBC: Periodic boundary condition
EAM: Embedded-atom method
L-J: Lennard-Jones
FCC: Face center cubic
HCP: Close-packed hexagonal
BCC: Body centered cubic
ICO: Icosohedral
SGB: Similar-to-grain boundary
SSD: Subsurface damage
